# T2-based temperature monitoring in abdominal fat during MR-guided focused ultrasound treatment of patients with uterine fibroids

**DOI:** 10.1186/s40349-015-0036-5

**Published:** 2015-09-11

**Authors:** Eugene Ozhinsky, Maureen P. Kohi, Pejman Ghanouni, Viola Rieke

**Affiliations:** Department of Radiology and Biomedical Imaging, University of California San Francisco, 185 Berry Street, Suite 350, Box 0946, San Francisco, CA 94143 USA; Department of Radiology, Stanford University, Stanford, CA USA

## Abstract

**Background:**

Near-field heating is a potential problem in focused ultrasound treatments, as it can result in thermal injury to skin, subcutaneous fat, and other tissues. Our goals were to determine if T2-based temperature mapping could be used reliably to measure near-field heating in adipose tissue and whether it is practical to perform such mapping during focused ultrasound treatments.

**Methods:**

We investigated the dependence of T2 on temperature in ex vivo adipose tissue at 3T using a double-echo fast spin echo (FSE) sequence. We implemented and evaluated the T2-based temperature mapping technique in the adipose tissue of two healthy volunteers. Finally, we applied the technique during magnetic resonance-guided focused ultrasound (MRgFUS) treatments to measure near-field heating in eight patients with uterine fibroids.

**Results:**

Calibration experiments in porcine adipose tissue determined a temperature coefficient of 6.16 ms/°C during heating and 5.37 ms/°C during cooling. The volunteer experiments demonstrated a strong correlation between the skin temperature and T2-based temperature measurements in the fat layer. During the treatments of patients with uterine fibroids, we observed a measurable change in the T2 of fat tissue within the path of the ultrasound beam and a temperature increase of up to 15 °C with sustained heating of more than 10 °C.

**Conclusions:**

Our results demonstrate the feasibility and importance of monitoring near-field heating in fatty tissues. The implementation of near-field monitoring between sonications can shorten treatments by reducing the cooling time. It can help improve safety by avoiding excessive heating in the near field.

## Background

Magnetic resonance-guided focused ultrasound (MRgFUS), also known as high-intensity focused ultrasound (HIFU), is a promising non-invasive technique that is commercially available for the treatment of symptomatic uterine fibroids [1]. The extracorporeal transducer delivers high-intensity ultrasonic energy through the skin, subcutaneous fat, muscle, and myometrium before it reaches the focus in the fibroid, with some absorption of ultrasound energy in the near field and subsequent tissue heating. Near-field heating is a potential problem in focused ultrasound treatments as it can result in skin burns and necrosis of healthy tissue. While each individual sonication causes only a moderate temperature rise in the near field, extended high-power sonications can lead to a significant cumulative thermal dose and cause damage to the tissue outside the target area [2].

During a fibroid treatment, proton resonance frequency (PRF) shift thermometry is used to monitor the heating during treatment due to its linearity and tissue-type independence [3]. However, while effective in water-based tissues, including muscle, myometrium, and the fibroid itself, PRF thermometry cannot detect temperature changes in abdominal fat [4]. In addition, PRF thermometry is very sensitive to magnetic field drift, respiration-induced field changes, and misregistration due to tissue movement or swelling [5] and, therefore, is difficult to reliably perform over long periods of time.

The manufacturers of commercial focused ultrasound systems have implemented various strategies to maximize treatment speed while avoiding tissue damage in the near field [6, 7]. For example, the Philips Sonalleve system performs volumetric ablation using treatment cells of various sizes by steering the focal spot along concentric circles and utilizes direct skin cooling [8]. The InSightec ExAblate system uses shorter duration sonications with smaller sonication spots. In addition, it supports elongated sonication spots, where the focal point travels along the axis of the transducer. Avoiding overlap of the beam path in successive sonications also effectively reduces heating in the near field. Due to the difficulty in direct measurement of near-field heating, the amount of cooling time for a specific sonication is calculated based on models [7] but does not take into account the variation of anatomical features, such as the composition and thickness of the near-field tissue layers, which can differ substantially between patients.

Relaxation time-based MR thermometry methods are more robust to the local field changes and can be used in adipose tissue. Graham et al. have shown the dependence of T1 and T2 on temperature in rabbit muscle [9]. Gandhi et al. presented the change of relaxation times with heating in bovine adipose tissue [10]. Recently, Baron et al. presented a technique to use T2 mapping to measure near-field heating in adipose tissue at 1.5 T and demonstrated its application in a Philips focused ultrasound system (Sonalleve, Philips Healthcare, Vantaa, Finland) [11]. They found that reversibility and linearity of the T2 temperature dependence of adipose tissue from 25 to 45 °C allowed for the monitoring of the temperature in the subcutaneous adipose tissue layers of in vivo pigs and human subjects.

The primary goal of this study was to investigate near-field heating in patients treated with the ExAblate 2100 uterine fibroid system (InSightec, Tirat Carmel, Israel) using a 3T MRI scanner. Next, we determined if T2-based temperature mapping could be used reliably to measure near-field heating in adipose tissue and whether it is practical to perform such mapping during focused ultrasound treatments. Our final goal was to evaluate the safety of the current treatment protocols and assess if accurate measurement of near-field heating in adipose tissue could lead to shorter treatments while preventing injury in healthy tissues.

## Methods

### Calibration experiments

**Fig. 1:**
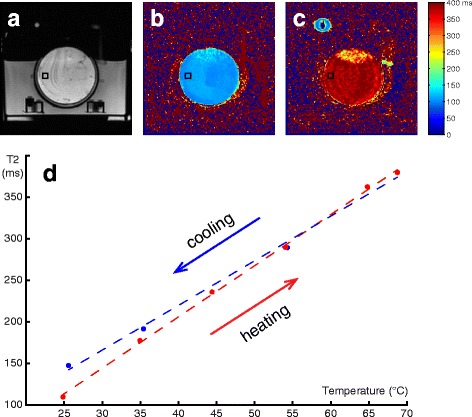
**a** FSE image of the petri dish containing porcine adipose tissue (no water suppression to visualize the setup). T2 maps of the tissue at 25 °C (**b**) and at 70 °C (**c**). **d** Plot of T2 versus temperature for porcine adipose tissue during heating (*red*) and cooling (*blue*), acquired with TE = 35/182 ms and water suppression

A petri dish was filled with porcine adipose tissue (Fig. 1) and placed in an insulated cylindrical container through which deionized water was circulated. The temperature of the circulating water was controlled by a temperature-regulated circulating water bath (Polystat R6L, Cole-Parmer, Vernon Hills, IL). The heat exchangers were placed in this temperature-regulated water bath to control the temperature of the water flow to the chamber in the MRI. The temperature within the sample and in the circulating water was monitored with fiber optic sensors (Luxtron, LumaSense Technologies, Santa Clara, CA). We determined the time necessary for temperature equilibration in the sample by measuring how long did it take for the readings of the probes to converge after a 10 °C change in the circulating water temperature.

T2 measurements were performed in a 3T MRI scanner (MR750W, GE Healthcare, Waukesha, WI) using a double-echo fast spin echo (FSE) sequence with and without water suppression with echo time (TE) of 35/182 and 28/147 ms, repetition time (TR) of 1500 ms, echo train length (ETL) of 40, field of view (FOV) of 12 cm, 128 × 128 matrix size, 8-mm slice thickness, and 15-s acquisition time. Water suppression was achieved via a spatial spectral pulse centered on the fat frequency. To ensure consistency, the frequency of the fat peak was measured with the manual pre-scan routine, and the same pre-scan parameter values were used throughout the experiment. Images were acquired during heating (25, 35, 45, 55, 65 and 70 °C) and subsequent cooling (55, 35 and 25 °C) after reaching thermal equilibrium. T2 maps were generated with an exponential fit for two data points [11].

### Healthy volunteers

T2 mapping for temperature measurement in subcutaneous fat was evaluated in two healthy volunteers. To measure the temperature on the surface of the skin, fiber optic probes (Luxtron, LumaSense Technologies, Santa Clara, CA) were attached to the skin on the back (volunteer 1) and abdomen (volunteer 2) of the volunteers who were positioned in the scanner supine and prone, respectively. Cooling and heating was performed with commercially available thermal pads (Hot/Cold Beads, Reusable Multi-Purpose Cold Compress, Walgreens, Deerfield, IL) placed on the skin in the region of the fiber optic probes. To prevent direct measurement of the heating pad, donut-shaped fiducial markers (diameter 15 mm, IZI Medical Products, Owings Mills, MD) were placed over the tips of the fiber optic probes. Images were acquired at baseline temperature, after cooling with a cold pad placed on the skin for several minutes and after heating with a hot pad.

T2 maps were acquired with a 3T MRI scanner (MR750W, GE Healthcare, Waukesha, WI) using a double-echo FSE sequence with water suppression (TE = 36/188 and 35/181 ms, TR = 1500 ms, ETL = 40, FOV = 32 cm, 256 × 128 and 128 × 128 matrix size, reconstructed to 256 × 256, 10-mm slice thickness, 15-s acquisition time). A Gaussian filter (size 10 × 10 px, standard deviation 5 px) was applied to the T2 maps in order to reduce noise.

### Patients

To evaluate the sequence in a clinical setting, fat temperature mapping was performed during clinical uterine fibroid treatments in eight patients using the ExAblate 2100 system (InSightec, Israel). All subjects provided informed consent as approved by the Institutional Review Boards (UCSF Committee on Human Research and Stanford University Institutional Review Board). T2 thermometry of abdominal fat was acquired at several time points during the treatment between sonications in a single coronal oblique slice. Imaging was performed using the double-echo FSE sequence with water suppression (TR = 1500 ms, TE = 35–41/181–197 ms, ETL = 40, FOV = 20–32 cm, 128 × 128 and 256 × 256 matrix size, reconstructed to 256 × 256, 10-mm slice thickness, 15–24-s acquisition time). During each sonication, the temperature was monitored with a PRF thermometry sequence as part of the standard clinical protocol.

At the end of the procedure, MRgFUS treatment summary files containing the parameters of each sonication (acoustic energy, focal spot position in patient coordinates, transducer tilt angles) were saved along with the thermometry images and transferred to an offline computer for processing. The T2 maps were generated from the acquired images using Matlab (Mathworks, Natick, MA). To reduce noise, a Gaussian filter (size: 10 × 10 px, st. dev. 5 px) was applied to the reconstructed T2 maps.

Maps of the temperature change since the baseline acquisition and between groups of sonications were generated using the calibration data from the ex vivo experiment. Intersections of the ultrasound beam axes and the T2 imaging slices were calculated using the sonication parameters from the treatment summary files and overlaid on the generated temperature maps. We plotted the adipose tissue temperature for the locations that experienced the highest temperatures along with the energy of the individual sonications to visualize the effect of ultrasound energy deposition over time using the time stamps of the MR images and the treatment summary files.

## Results

### Calibration experiments

Figure 1b, c shows examples of T2 maps of an adipose tissue sample at 25 and 70 °C. The T2 values within a 10 × 10 pixel ROI (black square on Fig. 1a) versus the temperature of the water bath at equilibrium are plotted in Fig. 1d. The fiber optic probe in the sample failed during the fourth cycle of heating. For the subsequent measurements, we relied on the probe in the circulating water and waited the previously estimated amount of time (~20 min) for the temperature within the sample to equilibrate. The T2 values in the fat sample increased linearly with heating but followed a slightly different slope during cooling.

Table 1 shows the linear regression coefficients of T2 versus temperature in ms/°C for the different acquisition parameters. The temperature coefficients during cooling were consistently smaller than during heating by 10, 13, and 12 %, respectively. There was approximately a 25 % difference between the measurements with and without water suppression. The difference in the T2/temperature coefficients between the two sets of echo times was smaller for heating (4 %) than for cooling (6 %).

**Table 1:** Relationship between T2 and temperature (ms/°C) for porcine adipose tissue, acquired with three protocols

	Heating	Cooling
Water suppr. TE = 28/147	6.41	5.74
Water suppr. TE = 35/182	6.16	5.37
No water suppr. TE = 35/182	4.64	4.10

### Healthy volunteers

**Fig. 2:**
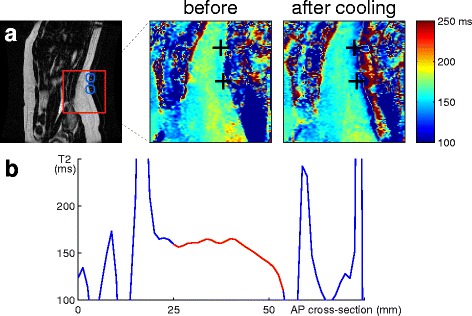
**a** Sagittal T2 mapping slice (volunteer 1) with the locations of the fiber optic probes before and after cooling of the area. Locations of fiber optic probes marked with *crosses*. **b** Cross-section of the T2 map at the level of the superior probe at the end of the cooling period. The fat layer is highlighted in *red*. The T2 maps show decreased T2 values, but only in the outer layer of fat near the skin

Fat temperature mapping was tested in two healthy volunteers. Figure 2 shows the propagation of the temperature change through the fat layer in the back of volunteer 1. The sagittal T2 maps (Fig. 2a) before cooling of the area and after cooling show the change in T2 values, but only in the outer layer of fat near the skin. The cross-section (Fig. 2b) shows the steep decrease in T2 values in the pixels of the fat area near the skin due to the cooling. The T2 decrease diminished within the fat layer with increasing distance to the skin. At about 10-mm depth in the fat layer, the temperature remained at body temperature throughout the experiment.

**Fig. 3:**
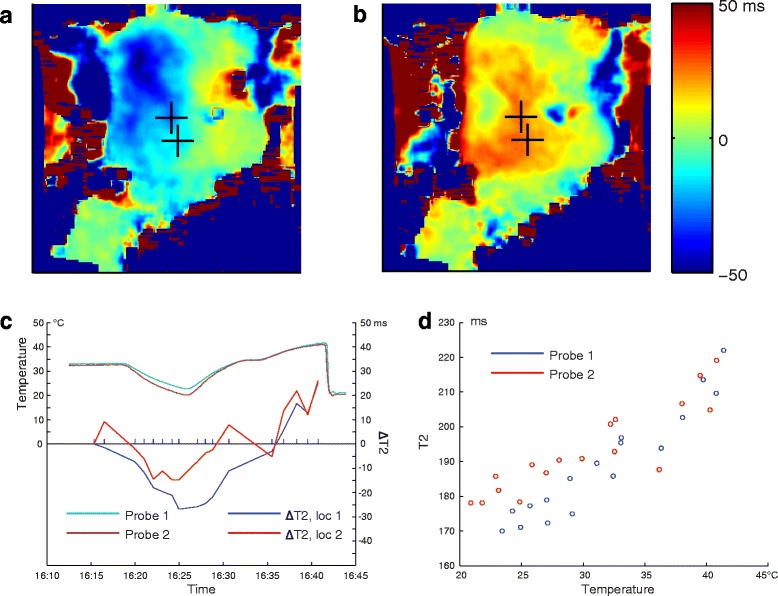
Coronal maps of T2 difference from baseline of abdominal fat layer (volunteer 2) after cooling (**a**) and heating (**b**). **c** Temperature, recorded by the probes and the corresponding change in T2 for volunteer 1 during cooling and heating. **d** Relationship between temperature and T2 at the locations of the probes. The temperature change in the middle of the fat layer, where T2 maps were acquired, was likely smaller than on the surface due thermal isolating properties of the fat layer

Figure 3 shows the coronal maps of the T2 difference from baseline of the abdominal fat layer in volunteer 2 after cooling (Fig. 3a) and heating (Fig. 3b). T2 values decrease with cooling of the tissue and increase during tissue heating. Figure 3c shows the temperatures, measured by the two fiber optic probes, overlaid with the calculated T2 change values in the locations closest to the probes. There was a strong correlation between T2 values in subcutaneous fat and fiber optic measurements (*R*^2^ = 0.91 and 0.8 for the first and second sensor locations, respectively). Quantitative measurement of the relationship between T2 and temperature was not possible in this in vivo setting without the ability to embed the fiber optic sensors directly into the tissue.

### Patients with uterine fibroids

**Fig. 4:**
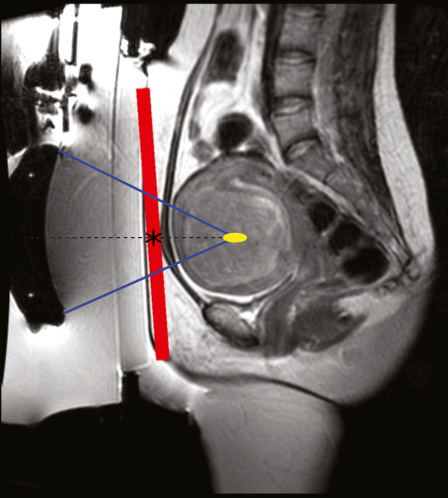
Sagittal image of the MRgFUS setup showing the uterine fibroid, focused ultrasound transducer within the oil bath, and coupling gel pad. Placement of the T2 mapping slice (*red*), the transducer axis (*dotted line*), and intersection of the axis and the slice (*asterisk*)

This technique was used to acquire thermometry data during the treatment of the eight patients with uterine fibroids (Table 2). Each treatment consisted of 57–149 sonications with acoustic energy of up to 5700 J and duration of 20 s per sonication. Near-field temperature was measured in six patients. In patient 1 and patient 7, it was not possible to quantify near-field heating due to patient repositioning during the treatment and patient motion, respectively. Figure 4 shows a sagittal image of the uterus and fibroid, the focused ultrasound transducer of the ExAblate system within an oil bath, and a gel pad for acoustic coupling. The area of intersection of the center of the beam path with the T2 mapping slice depends on the position of the focal spot and the position and the tilt angle of the transducer, which will be a circle (no tilt) or oval (with tilt) of approximately 60-mm diameter (range approx. 30 to 75 mm).

**Table 2:** Treatment summary of patients with uterine fibroids with near-field temperature monitoring

Patient	# sonications	Tx duration	# T2 maps	Max temp.
1	70	6:04	12	–
2	57	2:42	13	15.75
3	149	8:18^a^	11	8.23
4	82	5:09	3	7.78
5	70	4:20	12	7.48
6	88	3:59	6	6.58
7	81	2:56	5	–
8	77	4:23	10	6.49

*Columns* patient number, number of sonications in the treatment, treatment durations (hours, from first to last sonication), number of T2 measurements, maximum temperature (°C), measured in subcutaneous fat.

^a^Long treatment time caused by multiple equipment failures

**Fig. 5:**
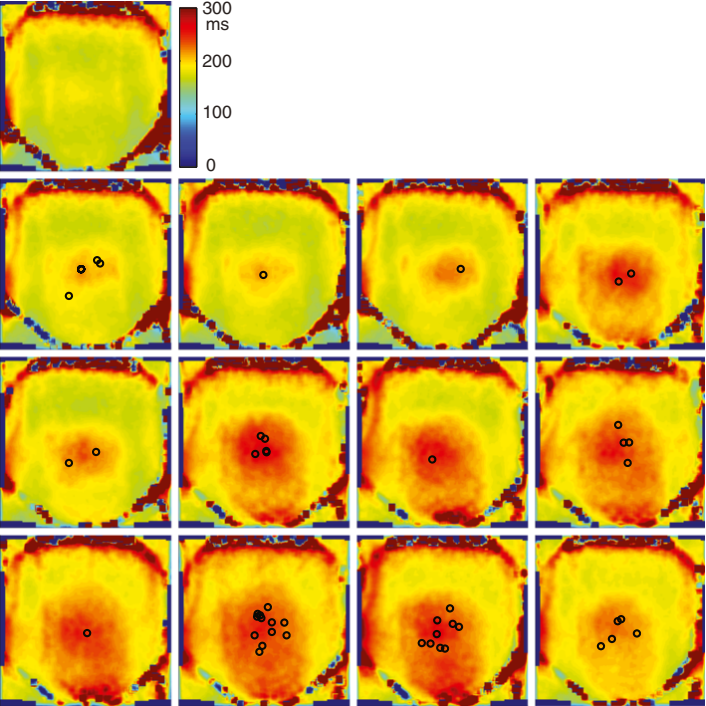
T2 maps in the coronal imaging slice over the course of the treatment of patient 2. Intersections of the beam axes and the slice (asterisk in Fig. 4) are shown as *circles*. There was a measurable change in the T2 of fat tissue in the path of the ultrasound beam

During the treatments, we observed a measurable change in the T2 of fat tissue in the path of the ultrasound beam. Figure 5 shows the T2 maps in the coronal imaging slice over the course of the treatment of patient 2. The upper image shows the baseline T2 map before the treatment. Over the course of this treatment, the T2 values in the abdominal fat layer increased and the area of increased T2 values grew larger.

**Fig. 6:**
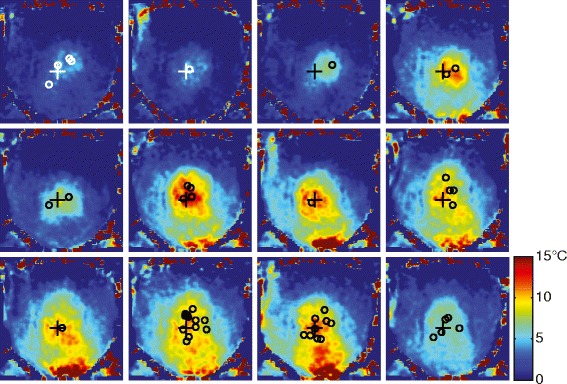
Temperature change from the baseline in the same patient. Intersections of the beam axes and the slice are shown as *circles*, location of the measurement as “*cross*”

Figure 6 shows the total change in temperature during the same treatment. For these maps, the T2 difference from the baseline was calculated and converted to temperature according to the calibration coefficient of 6.16 ms/°C, as measured in the calibration experiment for the water-suppressed sequence with TE = 35/182 ms (Table 1). The measured temperature increase in the ultrasound beam path reached close to 15 °C. Some artifacts are seen at the inferior edge of the imaging slice, which are not due to a temperature increase, as the beam path did not traverse through that area.

**Fig. 7:**
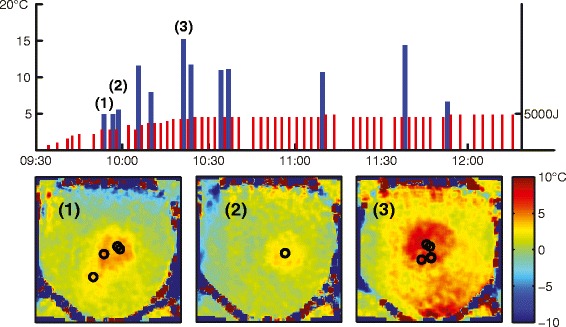
*Top*: measured temperature change from baseline (*blue bars*) for the location marked with a cross in Fig. 6 and energy of sonications (*red bars*) over the course of the treatment. *Bottom*: temperature change between subsequent measurements shows effects of groups of sonications and a single sonication (numbers indicated on timeline above)

Figure 7 provides an overview of the time course of the treatment depicted in Figs. 5 and 6. We observed temperature increases up to 15 °C and sustained heating of more than 10 °C for the duration of the treatment. Below the timeline are maps of the temperature difference between the consecutive T2 maps, which also show the number and location of the focused ultrasound sonications. It is clear from these different images that the areas of increased temperature following these sets of sonications matched the intersection of the US beam with the imaging slice.

**Fig. 8:**
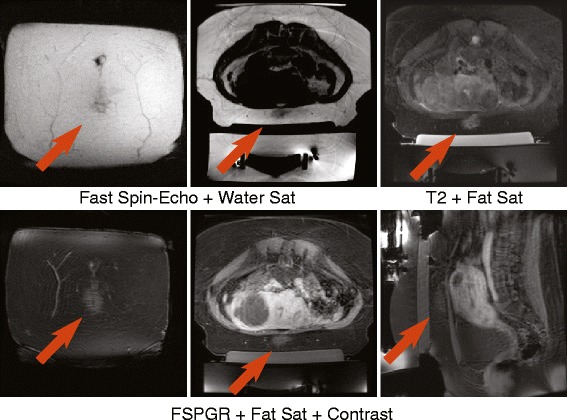
Post-treatment images of patient 5 showing adipose tissue damage. Coronal image through the abdominal fat layer (*upper* and *lower left*), axial (*upper middle* and *right*, *lower middle*) and sagittal (*lower right*) image through the middle of the treatment area

Of the eight treated patients, one has experienced near-field adipose tissue damage. Figure 8 shows the post-treatment images of patient 5 with a thick layer (approx. 34 mm) of subcutaneous fat. There was hypo-intensity in the FSE images acquired with water suppression and hyper-intensity in T2-weighted FSE images with fat saturation and post-contrast fast spoiled gradient echo (FSPGR) images. The final water-suppressed T2 map, acquired after the end of the treatment, showed no change in T2 values in the area. The patient reported tenderness and ecchymosis (bruising) on the surface of the abdomen in the area of the ultrasound beam path, which disappeared within a week after the treatment.

## Discussion

In this study, we have evaluated a technique for measuring the adipose tissue temperature change using T2 mapping. This technique has been developed for 1.5T and has been previously published [11]. After optimizing the sequence for 3T, we have calibrated the temperature dependence of T2 in porcine subcutaneous fat for temperatures between 25 and 70 °C at 3T, tested the technique in healthy volunteers, and measured near-field heating in adipose tissue for eight patients undergoing MR-guided FUS treatments for symptomatic uterine fibroids.

Our calibration experiments in porcine adipose tissue determined a temperature coefficient of 6.16 ms/°C during heating and 5.37 ms/°C during cooling for TE = 35/182 ms. Measuring the coefficients in images with a different echo time (TE = 28/147 ms) resulted in slightly higher values for both heating and cooling. For a temperature rise of 20 °C, this would result in an error of 1 °C. These results suggest that calibration of T2-based thermometry techniques should be done with parameters close to those used for temperature monitoring during the treatment of patients.

We also measured the coefficients in images with and without water suppression (using the same TE = 35/182 ms), which resulted in very different values. This may be due to the contribution of water spins to the measured T2 in the non water-suppressed acquisitions and/or suppression of a portion of the fat spins in the water-suppressed acquisition. In order to accurately suppress water, we manually adjusted the center frequency of fat in the manual pre-scan, as the auto pre-scan failed to produce consistent results between scans or did not work at all.

Earlier studies showed a reversible relationship between T2 and temperature for ex vivo adipose tissue [9, 12, 11]. Our calibration experiment covered a large range of temperatures (25–70 °C) and showed a slightly different temperature coefficient during heating and cooling. However, for the purpose of monitoring near-field heating, the range of expected temperatures would be much narrower (approximately 35–50 °C) and below the threshold of tissue damage. For this range, the errors in temperature estimation due to the hysteresis would be small. Future studies will look into the physical changes of the adipose tissue that could explain this hysteresis effect at higher temperatures. In addition, our experiments relied on ex vivo porcine fat tissue. Further studies are needed to quantify the relationship between relaxation parameters and temperature in in vivo or freshly excised human tissue, which was beyond the scope of this study.

The temperature calibration in the Baron’s study [11] was performed on a 1.5T MR scanner and resulted in an average coefficient of 5.2 ms/°C at 1.5T. As expected for a different field strength, our calibration experiments at 3T showed a different regression coefficient between T2 and temperature (see Table 1). We also found that this coefficient depends on acquisition parameters, such as water suppression and the choice of echo times. These findings indicate that calibration should be performed with the same parameters as the ones used during the treatment in order to get the most accurate temperature measurements.

The volunteer experiments demonstrated a strong correlation (*R*^2^ = 0.91 and *R*^2^ = 0.8) between the skin temperature measured with two fiber optic probes and T2-based temperature measurements in the fat layer close to the skin. However, a quantitative comparison was not possible in this volunteer experiment, as the sensors would need to be embedded within the fat layer. It is also noteworthy that trying to change the temperature deep within the subcutaneous fat layer was not possible by simply placing a hot or cold pad on the outside, due to the low thermal conductivity of fatty tissue. It is important to consider this insulation in the abdominal fat layer during a focused ultrasound treatment. While active cooling of the skin is very effective for preventing skin burns, it is likely to only affect the very outer layer (<10 mm) of the subcutaneous fat and may not prevent thermal dose damage in deeper tissues.

Our results demonstrate the feasibility and importance of monitoring near-field heating in fatty tissues using T2 mapping. During the long duration of fibroid treatments with the ExAblate system, near-field heating can increase temperatures in the adipose tissue substantially and result in a cumulative thermal dose that can potentially cause tissue necrosis [13, 14]. In one of the patient treatments, we observed adipose tissue damage in the area that received a significant amount of heating based on the T2 mapping. Hyper-intensity on the fat-suppressed and post-contact images together with hypo-intensity on the water-suppressed images suggested liquid accumulation in the tissue and was consistent with ecchymosis (bruising), reported by the patient post-treatment. The water-suppressed T2 maps did not show residual T2 change in that area at the end of the treatment. The longer term effects of adipose tissue damage following treatments with focused ultrasound remain to be studied.

Accurate monitoring of near-field heating should enable the physicians and equipment manufacturers to find optimized treatment strategies that avoid continuously heating the same region of near-field tissue. Implementing near-field monitoring between sonications would allow to reduce the cooling times and result in shorter treatments and more complete ablation of the target region. At the same time, it would improve safety by requiring longer cooling times when the near-field temperature reaches a certain threshold.

The limitations of the current study include relying on calibration data in ex vivo animal tissue and lack of external reference temperature measurements within the tissue in patient and volunteer studies. All experiments used the same T2-mapping protocol, but due to the variation in prescription, the actual parameters, such as echo times, varied slightly between the subjects and were presented as ranges in this paper. Our calibration experiment, which tested sequence parameters much further outside the range used in the patients, still showed only a small effect on the temperature measurement. Since the actual acquisition parameters may be different than those prescribed in the protocol, it is important to use the actual values recorded in the image headers for reconstruction of T2 maps.

Measurement of actual temperature at baseline would be necessary to accurately quantify the temperature reached during the treatment. More frequent measurements will allow better quantification of the cumulative thermal dose and the rate of cooling following the sonication.

## Conclusions

Our data show the feasibility and importance of measuring near-field heating in subcutaneous fat. We have quantified the temperature dependence of T2 in adipose tissue and shown that T2 mapping could be used to monitor the temperature increase during treatments of patients with uterine fibroids. We have also found that during treatment with the ExAblate system, near-field heating can reach high temperatures and cumulative thermal dose that may cause injury of adipose tissues. The implementation of near-field monitoring between sonications can shorten treatments by reducing the cooling time. It can help improve safety by avoiding excessive heating in the near field.

## Abbreviations

ETL: echo train length
FOV: field of view
FSE: fast spin echo
FSPGR: fast spoiled gradient echo
MRgFUS: magnetic resonance-guided focused ultrasound
PRF: proton resonance frequency
ROI: region of interest
TE: echo time
TR: repetition time
